# Skeletal Muscle ^31^P MR Spectroscopy Surpasses CT in Predicting Patient Survival After Liver Transplantation

**DOI:** 10.1002/jcsm.13635

**Published:** 2024-11-23

**Authors:** Denisa Kyselova, Irena Mikova, Petr Sedivy, Monika Dezortova, Milan Hajek, Jan Mares, Marek Tupy, Dana Kautznerova, Marek Kysela, Jiri Fronek, Julius Spicak, Pavel Trunecka

**Affiliations:** ^1^ Department of Hepatogastroenterology Institute for Clinical and Experimental Medicine Prague Czech Republic; ^2^ Institute of Physiology, First Faculty of Medicine Charles University Prague Czech Republic; ^3^ Department of Diagnostic and Interventional Radiology Institute for Clinical and Experimental Medicine Prague Czech Republic; ^4^ Department of Data Science Institute for Clinical and Experimental Medicine Prague Czech Republic; ^5^ Department of Transplantation Surgery Institute for Clinical and Experimental Medicine Prague Czech Republic; ^6^ Department of Anatomy, Second Faculty of Medicine Charles University Prague Czech Republic; ^7^ First Faculty of Medicine Charles University Prague Czech Republic

**Keywords:** ^31^P MR spectroscopy, liver transplantation, myosteatosis, sarcopenia, skeletal muscle

## Abstract

**Background:**

Skeletal muscle alterations are associated with higher mortality and morbidity in patients with liver cirrhosis. Assessing these changes seems to be a promising method for identifying patients at a high risk of poor outcomes following liver transplantation (LT). This is particularly important given the current global shortage of organ donors. However, evidence of the impact of these alterations on the prognosis of patients undergoing LT is inconclusive. The aim of our prospective study was to evaluate the impact of skeletal muscle changes, reflected in sarcopenia, myosteatosis and metabolic changes in the calf muscles, on perioperative outcomes and long‐term survival after LT. We also sought to determine the posttransplant evolution of the resting muscle metabolism.

**Methods:**

We examined 134 adult LT candidates. Of these, 105 underwent LT. Sarcopenia and myosteatosis were diagnosed by measuring the skeletal muscle index and mean psoas muscle radiation attenuation, respectively, which were obtained from computed tomography (CT) scans taken during pretransplant assessment. Additionally, patients underwent ^31^P MR spectroscopy (MRS) of the calf muscles at rest before LT and 6, 12 and 24 months thereafter. The median follow‐up was 6 years.

**Results:**

Patients with abnormal ^31^P MRS results and CT‐diagnosed myosteatosis prior to LT had significantly worse long‐term survival after LT (hazard ratio (HR), 3.36; 95% confidence interval (CI), 1.48–7.60; *p* = 0.0021 and HR, 2.58; 95% CI, 1.06–6.29; *p* = 0.03, respectively). Multivariable analysis showed that abnormal ^31^P MR spectra (HR, 3.40; 95% CI, 1.50–7.71; *p* = 0.003) were a better predictor of worse long‐term survival after LT than myosteatosis (HR, 2.78; 95% CI, 1.14–6.78; *p* = 0.025). Patients with abnormal ^31^P MR spectra had higher blood loss during LT (*p* = 0.038), required a higher number of red blood cell transfusions (*p* = 0.006) and stayed longer in ICU (*p* = 0.041) and hospital (*p* = 0.007). Myosteatosis was associated with more revision surgeries following LT (*p* = 0.038) and a higher number of received red blood cell transfusion units (*p* = 0.002). Sarcopenia had no significant effect on posttransplant patient survival. An improvement in the resting metabolism of the calf muscles was observed at 12 and 24 months after LT.

**Conclusions:**

Abnormal ^31^P MRS results of calf muscles were superior to CT‐based diagnosis of myosteatosis and sarcopenia in predicting perioperative complications and long‐term survival after LT. Resting muscle metabolism normalized 1 year after LT in most recipients.

## 1 Introduction

The undisputed prognostic value of skeletal muscle alterations in predicting the outcomes of various diseases, including chronic liver disease, has been the subject of extensive research in recent years. Several studies have reported an association between the presence of sarcopenia (loss of muscle mass and/or function) or myosteatosis (accumulation of excess fat in muscle) and high mortality, morbidity and poor quality of life in patients with liver disease [1, 2, 3, 4, 5]. According to a recent meta‐analysis, the overall prevalence of sarcopenia among patients with cirrhosis is as high as 37.5% [4]. Additionally, the presence of sarcopenia is associated with an approximately twofold increase in the risk of death in patients with cirrhosis [4]. The prevalence of myosteatosis in patients with cirrhosis is greater than 50% [1]. The condition is also associated with worse survival and a higher risk of hepatic encephalopathy [6]. Moreover, myosteatosis has proven to be of even better prognostic value than skeletal muscle mass in estimating outcomes in transplantation settings [7].

Sarcopenia and myosteatosis are easily assessed by calculating either the skeletal muscle index (SMI), where SMI = L3 skeletal muscle area (cm^2^)/height (m^2^), or the average skeletal muscle radiation attenuation (SMRA). Both measurements are typically obtained from computed tomography (CT) images at the level of the third lumbar vertebra (L3) [3, 8].

Assessment of skeletal muscle mass is an important tool for identifying liver transplant (LT) candidates with a significantly high risk of posttransplant mortality, particularly given the current global shortage of organ donors. In response to this challenge, proposals have been made to modify the risk index by incorporating myosteatosis as a factor in this evaluation [7]. Despite the large body of data supporting the prognostic role of evaluating skeletal muscle changes in patients with cirrhosis, data published thus far on the impact of skeletal muscle changes on short‐ and long‐term outcomes after LT are inconsistent and mostly sourced from retrospective studies. Although some studies report that pretransplant myosteatosis, but not sarcopenia, could be an important posttransplant prognostic marker in patients undergoing LT, especially in the early postoperative period [7, 9], other studies have found no adverse clinical outcomes associated with myosteatosis [10]. Most of the studies that have correlated sarcopenia or myosteatosis with LT outcome in retrospective fashion have used CT imaging‐based techniques to calculate the SMI and SMRA as part of the routine pretransplant evaluation of candidates. However, these indicators do not provide insights into muscle function or muscle metabolism.

^31^P magnetic resonance spectroscopy (MRS) of the skeletal muscles is an established noninvasive method for assessing muscle energy metabolism [11]. The method is based on the quantitative analysis of ^31^P‐containing metabolites such as phosphocreatine, adenosine triphosphate (ATP), phosphodiesters and inorganic phosphate. Changes in the relative concentrations of these compounds can indicate the presence of certain diseases [12, 13, 14, 15] and provide further prognostic information on muscle health in the context of liver disease. Most ^31^P MRS studies focus on the quadriceps femoris or gastrocnemius muscles, which are conveniently positioned for ^31^P MRS examination [11]. Although the method is limited in that it requires specific MR equipment and an experienced MR spectroscopist to conduct data evaluation, it is currently the only noninvasive approach that allows skeletal muscle metabolism to be studied in vivo.

The aim of our prospective study was to explore the prognostic value of changes in muscle energy metabolism assessed by ^31^P MRS and muscle morphology (sarcopenia and myosteatosis) assessed by CT, in a cohort of LT candidates, with a focus on understanding the impact of these changes on short‐ and long‐term posttransplant outcomes. Additionally, we were interested in charting the evolution of resting skeletal muscle metabolism after LT. We hypothesized that changes in muscle energy metabolism assessed by ^31^P MRS would be more predictive of posttransplant outcomes than morphological muscle changes assessed by CT.

## 2 Methods

### 2.1 Study Design and Patient Population

This prospective study included 134 adult patients who were evaluated as candidates for LT at the Institute for Clinical and Experimental Medicine, Prague, Czech Republic, between May 2015 and August 2017. Patients with acute liver failure were excluded, as were patients who were unable to provide their informed consent or incapable of undergoing MR examination. For details of the study protocol, see Figure S1.

All patients provided their informed consent to participate in the study. The study conformed to the ethical guidelines of the 1975 Declaration of Helsinki and was approved by the joint ethics committee of the Institute for Clinical and Experimental Medicine and Thomayer University Hospital, Prague, Czech Republic.

### 2.2 Clinical, Laboratory and Outcome Data

Clinical and laboratory data were obtained from patients at the time of LT assessment, serving as the baseline. In patients undergoing LT, clinical and laboratory data were collected 6, 12 and 24 months after the procedure. The original MELD score without adjustment for sodium was used. Exception points for specific indications were not allocated. The 2009 International Diabetes Federation (IDF) criteria for clinical diagnosis of metabolic syndrome were used. Liver stiffness was measured using two‐dimensional shear wave elastography (2D‐SWE). The following data on short‐term LT outcomes were recorded: duration of liver surgery, blood loss during the procedure, number of red blood cell transfusion units used, number of revision surgeries, length of intensive care unit (ICU) stay and length of hospital stay. Data on the long‐term survival of patients after transplantation were recorded up until 15 October 2022. The median follow‐up in the transplanted population was 6 years.

### 2.3 Sarcopenia and Myosteatosis Assessment by CT

Multidetector CT was performed using a 256‐row CT scanner (Somatom Sensation, Siemens Healthineers) to evaluate sarcopenia and myosteatosis. CT was calibrated for regular clinical use; scanning was performed using a tube voltage of 120 kV and automatic tube current modulation. Measurements were obtained from the last CT examination carried out before surgery. The median time between the CT scan and LT was 98 days (IQR, 52–158) in the transplanted population.

Cross‐sectional scans at the level of the L3 vertebra were used, segmenting the iliopsoas muscle and abdominal skeletal muscles. To diagnose sarcopenia, the skeletal muscle area comprising the iliopsoas, erector spinae, quadratus lumborum, transversus abdominis, obliquus externus, obliquus internus and rectus abdominis muscles was measured in cm^2^ using VOI freehand in Syngo.via (Siemens, Germany). The SMI (cm^2^/m^2^) was calculated by dividing the skeletal muscle area by the square of the patient's height. Sarcopenia was defined by established cutoff values for patients with cirrhosis, namely, an SMI < 50 cm^2^/m^2^ for men and an SMI < 39 cm^2^/m^2^ for women [3, 15, 16]. Myosteatosis was diagnosed based on the average density of the iliopsoas muscle using psoas muscle radiation attenuation (PMRA) measured in Hounsfield units (HU). For patients with a BMI of < 25 kg/m^2^, a cutoff value of < 41 HU was used; for patients with a BMI of ≥ 25 kg/m^2^, a cutoff value of < 33 HU was used [8]. Illustrative CT images of sarcopenic, nonsarcopenic, normal and low muscle density are shown in Figure S2.

### 2.4 Resting Skeletal Muscle Metabolism Assessed by ^31^P MR Spectroscopy

MR examination was performed using a 3 T whole‐body MR scanner (Magnetom Trio, Siemens Healthineers, Erlangen, Germany) equipped with a dual‐channel ^31^P/^1^H surface coil with a diameter of 11 cm (Rapid Biomedical, Rimpar, Germany). All subjects were examined in a supine position with the coil fixed underneath the calf. The positioning of the coil was verified using a standard localizer sequence.

^31^P MR spectra were acquired at rest using a nonlocalized free induction decay (FID) sequence, covering most of the ^31^P MR signal from the gastrocnemius and soleus muscles. The following parameters were applied for the sequence: echo time (TE*) = 0.4 ms, repetition time (TR) = 15 s, 16 acquisitions, matrix size = 1024, acquisition time = 4 min. Magnetic field homogeneity was optimized manually through localized shimming of the water signal.

^31^P MR spectra were analysed using an advanced method for accurate, robust and efficient spectral fitting (AMARES), which is a time‐domain fitting algorithm based on prior knowledge. The analysis was performed using the jMRUI 5.0 software package (http://www.jmrui.eu/) [17, 18]. Lorentzian line shapes were applied to fit the following singlets: phosphocreatine (PCr), inorganic phosphate (Pi) and phosphodiester signals. Peaks in ATP were fitted as two doublets (αATP and γATP) and a triplet (βATP). The relative chemical shift of Pi and PCr (δ in ppm) was used to calculate the intracellular pH according to the following equation: pH = 6.75 + log[(δ − 3.27)/(5.63 − δ)] [19].

The cross‐section of the gastrocnemius muscle was determined from a transverse MR image of the calf muscle obtained using a fast low‐angle shot (FLASH) sequence (parameters: TE = 5 ms, TR = 20 ms, matrix = 312 × 384, slice thickness = 6 mm, 15 slices). The muscle was subsequently manually segmented in ImageJ software (https://imagej.nih.gov/) [20].

**FIGURE 1:**
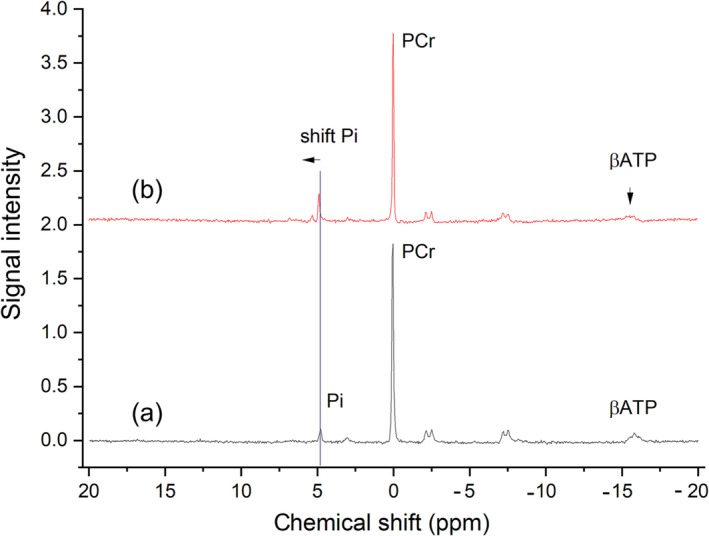
(a) Normal and (b) abnormal ^31^P MR spectra of LT candidates. A relatively lower signal intensity of βATP and a shift in the Pi signal were observed in abnormal spectra. Criteria of abnormality: βATP/P_tot_ < 0.074 or pH at rest > 7.045 (see Section 2.4).

Two cutoff parameters were used to distinguish abnormal resting muscle metabolism: a ratio of the βATP signal intensity to the sum of all ^31^P metabolite intensities (βATP/P_tot_) < 0.074, representing the deterioration in the energy status of muscle cells, or a resting intramyocellular pH value of > 7.045, serving as an indicator of overall muscle cell homeostasis. The specific cutoff values for changes in ATP and pH were chosen based on our previously published results obtained from healthy controls who underwent the same examination protocol [21]. These values were calculated according to the following formulas: cutoff βATP/P_tot_ = mean βATP/P_tot_ − standard deviation βATP/P_tot_; cutoff pH = mean pH + standard deviation pH. Normal and abnormal ^31^P MR spectra are shown in Figure 1.

Muscle strength (MSt) was determined by calculating the average of five maximal voluntary contractions measured in newtons (N) using a foot dynamometer.

MR examinations were performed at baseline during the pretransplant assessment and, in the case of the transplanted population, at 6, 12 and 24 months after LT. The median time between baseline MR and LT was 3.6 months (IQR, 1.97–5.57).

### 2.5 Statistical Analysis

Both the population of transplanted patients and the entire population of LT candidates were analysed based on available data; no data imputations were performed. The quantities of missing and available data are presented in the text and tables.

For survival analysis, the Kaplan–Meier estimator of survival function was used to obtain time‐point estimates for 5‐year posttransplant survival; between‐group differences were tested using the log‐rank test. The Cox proportional hazards model was fitted for both univariable and multivariable analysis. The predictors were preselected based on the background knowledge. The subset used in the multivariable model was chosen using the backward elimination method, iterated until the number of predictors decreased to less than one‐tenth of the number of observed events to avoid overfitting. The limited number of events observed did not allow for a higher ratio of events per predictor, which would have otherwise been advisable because of the postselection of predictors. Fisher's exact test was used to assess differences in proportions; the Wilcoxon rank‐sum test was used for scale variables. A standard alpha level of 5% was used to assess statistical significance; *p*‐values are presented without a multiple testing correction. All results other than the main outcomes must be interpreted as exploratory. In other words, there is a considerable chance that some of the statistically significant differences are false positives. Point estimates are presented with 95% confidence intervals (CIs). The analysis was performed using R software, version 4.2.2.

## 3 Results

### 3.1 Baseline Characteristics

Baseline demographic and clinical data of all patients included in the study are shown in Table 1. Of the 134 LT candidates, 74 were men (55.2%) with a median age of 59.6 years (IQR, 49.2–65.3). The median Child–Pugh score and model for end‐stage liver disease (MELD) were 9 (IQR, 7–10) and 14 (IQR, 11–17), respectively. The most common indications for LT in our cohort were alcoholic liver disease (ALD) (46 patients, 34.3%), cholestatic liver disease (primary biliary cholangitis (PBC), primary sclerosing cholangitis (PSC), PSC–autoimmune hepatitis (AIH) overlap syndrome) (23 patients, 17.2%), HCV (18 patients, 13.4%) and metabolic dysfunction–associated steatohepatitis (MASH) (14 patients, 10.5%). Hepatocellular carcinoma (HCC) was present in 34 LT candidates (32.4%). All indications for LT in our cohort are given in Table S1. Detailed tumour characteristics in patients transplanted for HCC are given in Table S2.

**TABLE 1:** Baseline demographic, clinical and laboratory data for LT candidates (*N* = 134) and the transplanted population (*n* = 105). Data are given as *N* (%) or the median (first to third quartiles).

Variable	Transplanted population (*n* = 105)	LT candidates (*N* = 134)	Reference range
Male sex	60 (57.1%)	74 (55.2%)	
Age (years)	59.8 (48.9, 65.6)	59.6 (49.2, 65.3)	
BMI (kg/m^2^)	26.6 (23.4, 29.2)	26.1 (23.2, 29.4)	
Hypertension	47 (44.8%)	58 (43.3%)	
Diabetes	49 (46.7%)	58 (43.3%)	
Metabolic syndrome	55 (52.4%)	70 (52.2%)	
Child–Pugh score	9 (7, 10)	9 (7, 10)	
MELD score	14 (11, 17)	14 (11, 17)	
Ascites	56 (53.3%)	72 (53.8%)	
Bilirubin (μmol/L)	39.5 (20.5, 69.8)	41.8 (20.9, 72.6)	3.4–20
Albumin (g/L)	29.5 (25.1, 35.5)	29.3 (25.0, 34.3)	36–45
INR	1.29 (1.18, 1.45)	1.31 (1.18, 1.46)	0.8–1.2
Glycemia (mmol/L)	4.9 (4.6, 5.5)	5.0 (4.6, 5.9)	3.6–5.59
HbA1c (%)	30 (25, 38)	30 (24, 38)	20–42
HOMA‐IR^a^	2.22 (1.47, 4.35)	2.47 (1.56, 4.62)	0.5–1.4
Triglycerides (mmol/L)^b^	0.88 (0.67, 1.16)	0.88 (0.66, 1.15)	0.5–1.69
Total cholesterol (mmol/L)^b^	3.6 (2.9, 4.5)	3.55 (2.73, 4.48)	2.9–5
LDL cholesterol (mmol/L)^b^	2.3 (1.9, 3.1)	2.3 (1.7, 2.9)	1.2–3
HDL cholesterol (mmol/L)^b^	0.79 (0.62, 1.02)	0.78 (0.58, 1.02)	1–2.1
Liver stiffness (kPa)^c^	36.0 (22.4, 47.7)	36.6 (26.2, 48.0)	
Sarcopenia^d^	49 (47.6%)	59 (44.7%)	
SMI (cm^2^/m^2^) M:F^d^	49 (42, 56):41 (34, 47)	50 (42, 56):41 (35, 46)	
Myosteatosis^d^	53 (51.5%)	70 (53%)	
PMRA (HU)^d^	25 (17, 31)	23.5 (15.8, 30.3)	
βATP/P_tot_ ^e^	0.083 (0.077, 0.089)	0.083 (0.076, 0.089)	
Resting intramyocellular pH^e^	7.018 (6.997, 7.036)	7.018 (6.997, 7.036)	
Abnormal ^31^P MR spectra^e^	32 (32.3%)	42 (33.6%)	

Abbreviations: BMI: body mass index, F: female, HbA1c: glycated haemoglobin, HDL: high‐density lipoprotein, HOMA‐IR: homeostatic model assessment of insulin resistance, INR: international normalized ratio, LDL: low‐density lipoprotein, M: male, MELD score: model for end‐stage liver disease score, MR: magnetic resonance, PMRA: psoas muscle radiation attenuation, SMI: skeletal muscle index, βATP/P_tot_: ratio of the βATP signal intensity to the sum of all ^31^P metabolite intensities.

^a^ Assessed only in 132 of the LT candidates and in 104 patients from the transplanted population.

^b^ Assessed only in 114 of the LT candidates and in 89 patients from the transplanted population.

^c^ Assessed only in 121 of the LT candidates and in 94 patients from the transplanted population.

^d^ Assessed only in 132 of the LT candidates and in 103 patients from the transplanted population.

^e^ Assessed only in 125 of the LT candidates and in 99 patients from the transplanted population.

Of the 134 LT candidates evaluated, 122 patients were placed on the waiting list and 105 underwent LT from deceased donors, a group we refer to here as the transplanted population (Figure S1). The median time between LT assessment and the actual listing or LT was 16 days (IQR, 9–30) and 3.6 months (IQR, 2.0–5.6), respectively. Whole LT, reduced graft LT and split graft LT were performed in 82, 9 and 14 cases, respectively. Of the 105 deceased donors, 73 were men (69.5%) with a median age of 52 years (IQR, 38.8–63) and a median BMI of 26.1 (IQR, 24.5–29.3).

### 3.2 Skeletal Muscle Characteristics and Long‐Term Survival After LT

**FIGURE 2:**
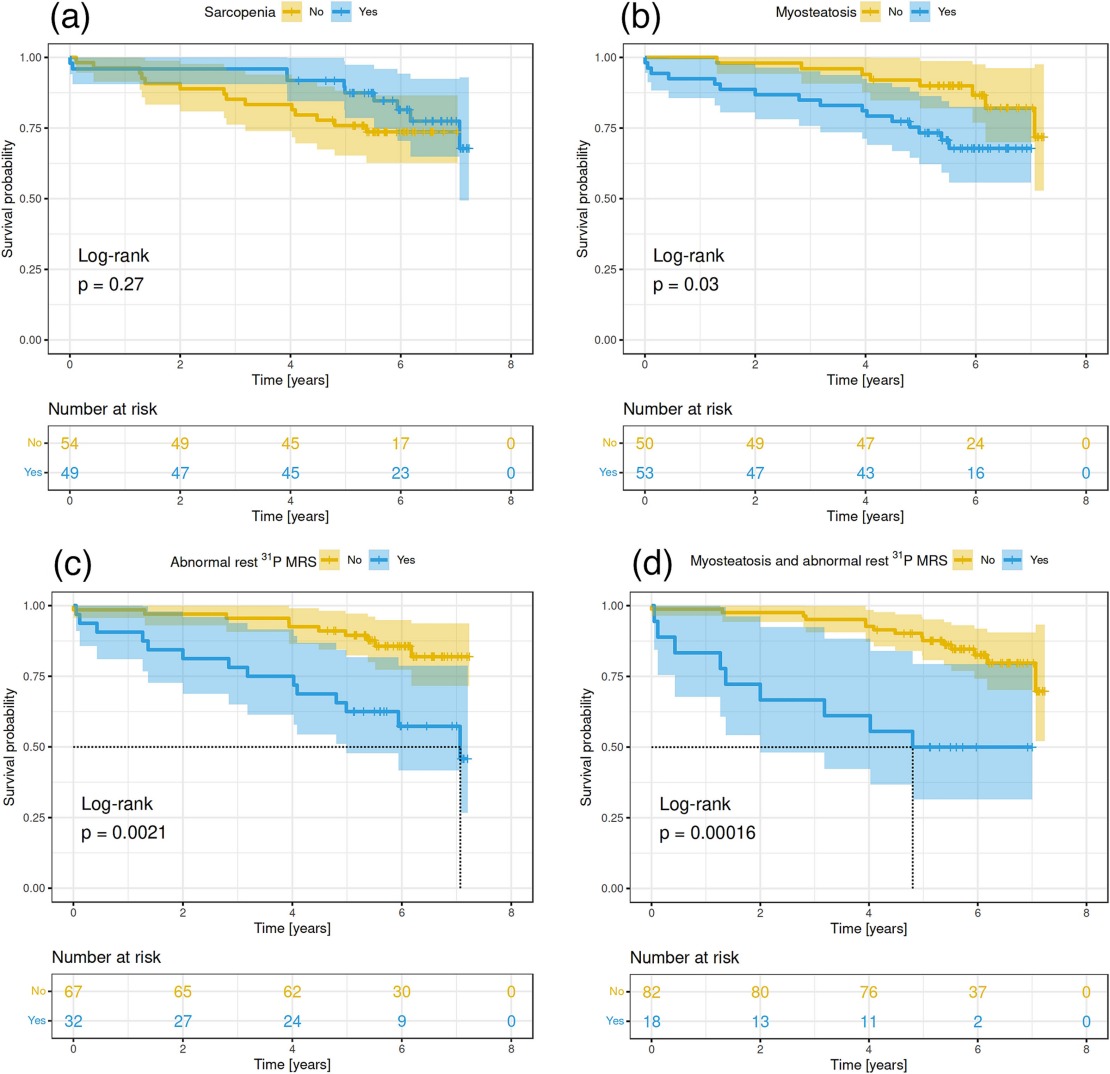
Probability of posttransplant survival in the transplanted population based on the presence of pretransplant (a) sarcopenia, (b) myosteatosis, (c) abnormal ^31^P MR spectra and (d) both myosteatosis and abnormal ^31^P MR spectra. Kaplan–Meier analysis (step functions) with 95% confidence intervals (semitransparent areas).

Results of CT‐based muscle examination or ^31^P MRS are presented in Table 1. We did not observe any significant difference in patient survival in relation to sarcopenia in the transplanted population or in LT candidates (*p* = 0.27 and *p* = 0.12, respectively; Figures 2a and S4A).

Patients with myosteatosis had worse long‐term posttransplant patient survival compared with patients with higher muscle density (HR, 2.58; 95% CI, 1.06–6.29; *p* = 0.03; Figure 2b). Worse long‐term survival was also observed when we analysed the impact of myosteatosis in the cohort of all LT candidates (HR, 2.01; 95% CI, 1.09–4.02; *p* = 0.023; Figure S4B). Likewise, patients with abnormal ^31^P MR spectra of the calf muscles exhibited significantly worse survival following LT, as indicated by both Kaplan–Meier analysis and the Cox model (HR, 3.36; 95% CI, 1.48–7.60; *p* = 0.0021; Figure 2c). Similarly, LT candidates with abnormal ^31^P MR spectra had worse survival outcomes (HR, 1.89; 95% CI, 1.01–3.53; *p* = 0.042; Figure S4C).

Patients with myosteatosis and abnormal ^31^P MR spectra had significantly worse 5‐year survival probability compared with patients without myosteatosis and normal ^31^P MR spectra (*p* = 0.025 and *p* = 0.004, respectively; Table S3). Patients with both myosteatosis and abnormal ^31^P MR spectra had a 5‐year survival probability of 50% (95% CI, 32.5–79.4) compared with 87.6% in all other cases (95% CI, 80.8–95.1; *p* = 0.002; Figure 2d).

There was no significant difference in the survival of patients with a full graft compared with a reduced graft or a split graft (HR, 0.52; 95% CI, 0.22–1.24; *p* = 0.14).

Based on multivariable analysis, both myosteatosis and abnormal rest ^31^P MR spectra were independent predictors of long‐term survival after LT (Table 2). Moreover, ^31^P MR spectra appeared to be a stronger predictor of long‐term survival after LT than myosteatosis.

**TABLE 2:** Univariable and multivariable models fitted to identify variables associated with long‐term survival in the transplanted population using the Cox proportional hazards model. Variables for the multivariable model were chosen using the backward elimination method, iterated until the number of predictors decreased to less than one‐tenth of the number of observed events to avoid overfitting. We registered 24 deaths in the combined model. HRs with corresponding 95% CIs and *p*‐values were calculated to determine whether the HRs differed significantly from 1.

		Univariable model		Multivariable model	
	*N*	HR (95% CI)	*p*	HR (95% CI)	*p*
Sarcopenia	103	0.63 (0.27–1.64)	0.28	‐‐—	‐‐—
Myosteatosis	103	**2.58 (1.06**–**6.29)**	**0.037**	**2.78 (1.14**–**6.78)**	**0.025**
Abnormal ^31^P MRS	103	**3.36 (1.48**–**7.60)**	**0.004**	**3.40 (1.50**–**7.71)**	**0.003**
Age (per year)	105	1.04 (0.99–1.09)	0.11	‐‐—	‐‐—
BMI (per point)	105	1.07 (0.99–1.16)	0.09	‐‐—	‐‐—
Metabolic syndrome	105	1.52 (0.66–3.47)	0.33	‐‐—	‐‐—
HOMA‐IR	104	1.02 (0.95–1.10)	0.52	‐‐—	‐‐—
MELD	105	0.92 (0.83–1.02)	0.11	‐‐—	‐‐—
Child‐Pugh score	105	0.98 (0.82–1.17)	0.85	‐‐—	‐‐—
HCC presence	105	2.30 (1.02–5.19)	0.045	‐‐—	‐‐—

Abbreviations: BMI: body mass index, CI: confidence interval, HCC: hepatocellular carcinoma, HOMA‐IR: homeostatic model assessment of insulin resistance, HR: hazard ratio, MELD score: model for end‐stage liver disease score, MRS: magnetic resonance spectroscopy, *p*: *p*‐value.

**FIGURE 3:**
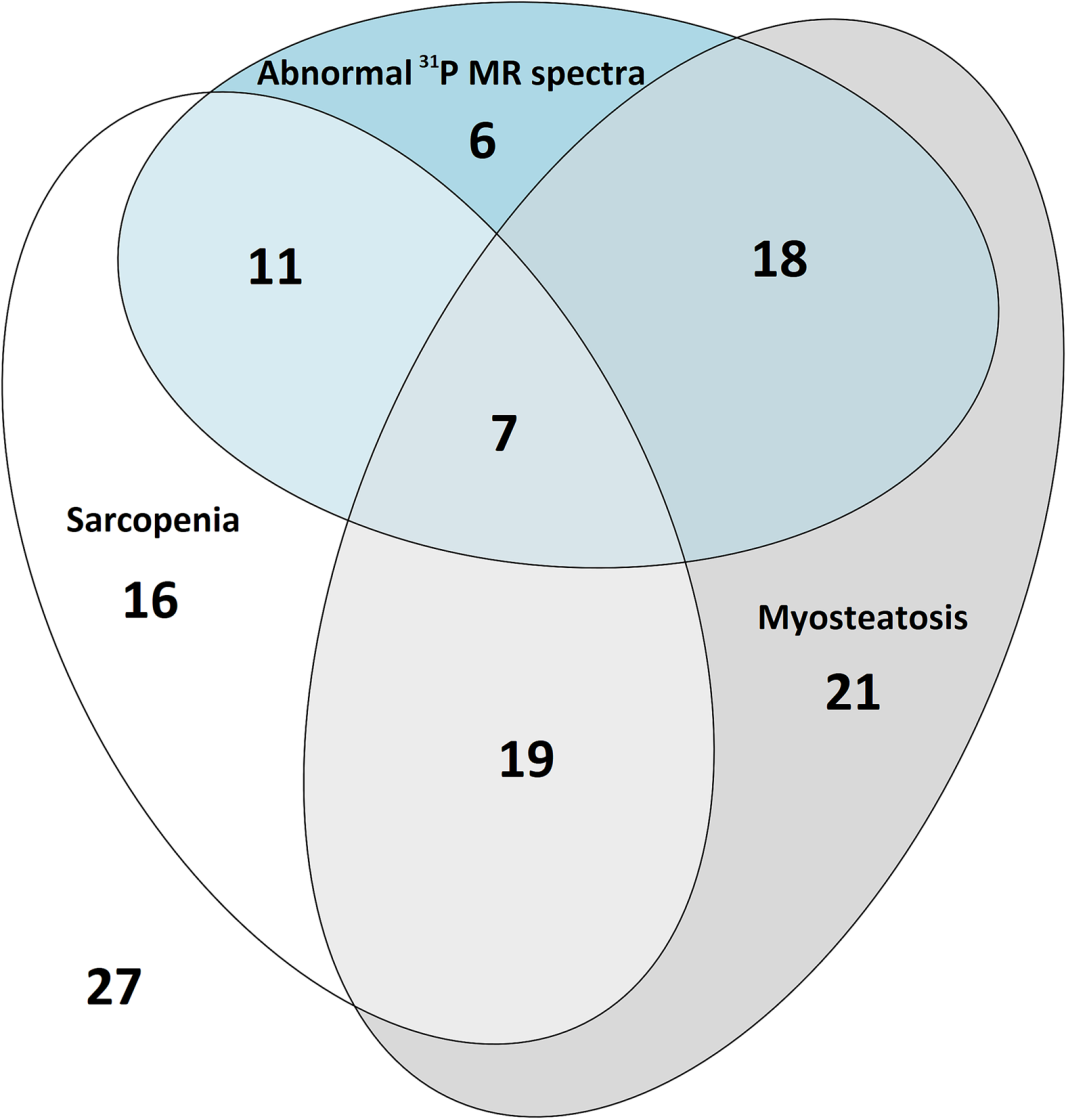
Euler diagram illustrating the relationships between the presence of sarcopenia, myosteatosis and abnormal ^31^P MR spectra.

We assessed correlations between parameters based on the ^31^P MR spectra of calf muscles at rest, SMI (sarcopenia) and PMRA (myosteatosis). Data are presented in Table 3 and scatterplots in Figure S3. We found a positive correlation between SMI and the ratio of the βATP signal to the sum of all ^31^P metabolites (*r* = 0.36; 95% CI, 0.18–0.52; Figure S3B). Otherwise, we detected no correlation between the presence of abnormal ^31^P MR spectra and myosteastosis or sarcopenia (*r* = 0.08; 95% CI, −0.12 to 0.27 and *r* = 0.02; 95% CI, −0.17 to 0.22, respectively). There was a positive correlation between the area of the gastrocnemius muscle, assessed by MR and the SMI, assessed by CT (*r* = 0.43; 95% CI, 0.27–0.58; Table 3). Patients with myosteatosis exhibited lower muscle strength compared with patients with normal muscle density (*p* = 0.049; Table 3). We did not find any correlation between sarcopenia and myosteatosis or between the SMI and PMRA (*r* = −0.04; 95% CI, −0.21 to 0.13 and *r* = −0.14; 95% CI, −0.36 to 0.07, respectively; Figure S3A). Relationships between the incidences of all three muscle characteristics (sarcopenia, myosteatosis and abnormal ^31^P MR spectra) in LT candidates are graphically depicted in Figure 3.

**TABLE 3:** MR parameters of calf muscles in patients with and without sarcopenia and with and without myosteatosis. Values were analysed for their correlations (*r*) with SMI (skeletal muscle index as a marker of sarcopenia, cm^2^/m^2^) or PMRA (psoas muscle radiation attenuation as a marker of myosteatosis, HU). The gastrocnemius muscle area (MGA), muscle strength (MSt), resting pH and signal intensity ratios of phosphocreatine (PCr), inorganic phosphate (Pi), ß‐adenosine triphosphate (ßATP) and phosphodiesters (PDE) to the sum of all signal intensities (P_tot_) are all given. Data are expressed as the median (first to third quartiles).

	With sarcopenia (*n* = 51)	Without sarcopenia (*n* = 56)	*p*	*r* with SMI (95% CI)	With myosteatosis (*n* = 55)	Without myosteatosis (*n* = 52)	*p*	*r* with PMRA (95% CI)
MGA (cm^2^)	18 (14, 21)	20 (18, 23)	**0.015**	**0.43 (0.27**–**0.58)**	19 (15, 22)	21 (16, 23)	0.23	0.02 (−0.39 to 0.28)
MSt (N)	390 (295, 435)	360 (283, 431)	0.60	0.24 (0.00–0.44)	330 (232, 418)	390 (313, 473)	**0.049**	0.16 (−0.05 to 0.34)
PCr/Pi	6.64 (5.87, 8.01)	6.88 (6.28, 7.76)	0.46	−0.05 (−0.23 to 0.13)	6.60 (6.14, 7.47)	6.98 (6.39, 8.31)	0.22	0.17 (−0.01 to 0.33)
PCr/P_tot_	0.493 (0.476, 0.514)	0.497 (0.482, 0.513)	0.61	−0.17 (−0.34 to 0.00)	0.497 (0.480, 0.514)	0.495 (0.477, 0.513)	0.82	0.13 (−0.04 to 0.27)
ßATP/P_tot_	0.079 (0.073, 0.086)	0.085 (0.079, 0.091)	**0.023**	**0.36 (0.18**–**0.52)**	0.083 (0.075, 0.087)	0.086 (0.077, 0.091)	0.17	0.15 (−0.20 to 0.38)
Pi/P_tot_	0.073 (0.064, 0.082)	0.072 (0.064, 0.078)	0.43	−0.04 (−0.21 to 0.14)	0.074 (0.066, 0.081)	0.072 (0.063, 0.078)	0.24	−0.15 (−0.34 to 0.12)
PDE/P_tot_	0.046 (0.040, 0.056)	0.047 (0.039, 0.058)	0.75	0.13 (−0.09 to 0.34)	0.046 (0.038, 0.054)	0.048 (0.045, 0.062)	0.063	−0.09 (−0.26 to 0.14)
pH	7.00 (6.84, 7.03)	6.98 (6.91, 7.02)	0.77	0.17 (−0.02 to 0.34)	6.98 (6.89, 7.02)	7.0 (6.85, 7.02)	0.90	0.00 (−0.18 to 0.20)

*Note:* SMI and PMRA values were obtained from 132 patients; MRS values (first column) were obtained from 125 patients.

Abbreviation: *r*: correlation coefficient.

### 3.3 Impact of Muscle Alterations on Perioperative Outcomes After Liver Transplantation

In addition to assessing the impact of muscle alterations on long‐term survival, we investigated the effect of sarcopenia, myosteatosis and changes in ^31^P MR spectra on perioperative outcomes in our cohort (Table 4). We did not observe any association between sarcopenia and short‐term outcomes. However, we did observe a statistically significant association between myosteatosis and increased resource utilization, including a higher number of intraoperative red blood cell transfusions (*p* = 0.043), postoperative red blood cell transfusions (*p* = 0.002), total red blood cell transfusions (*p* = 0.002) and a higher number of revision surgeries (*p* = 0.038). Patients with abnormal ^31^P MR spectra had longer ICU and hospital stays (*p* = 0.041 and *p* = 0.007, respectively), higher blood loss (*p* = 0.038) and a greater need for postoperative and total red blood cell transfusions (*p* = 0.012 and *p* = 0.006, respectively). Detailed results are presented in Table 4.

**TABLE 4:** Effect of sarcopenia, myosteatosis and abnormal ^31^P MR spectra on posttransplant resource utilization. Data are expressed as *N* (%) or the median (first to third quartiles).

	Sarcopenia	Without sarcopenia	*p*	Myosteatosis	Without myosteatosis	*p*	Abnormal ^31^P MRS	Normal ^31^P MRS	*p*
90‐day mortality	2/49 (4.1%)	1/54 (1.9%)	0.60	3/53 (5.7%)	0/50 (0.0%)	0.24	2/32 (6.3%)	1/67 (1.5%)	0.24
LT duration (min)	170 (145, 230)	174 (138, 244)	0.56	166 (136, 242)	175 (146, 228)	0.39	180 (146, 249)	170 (143, 233)	0.29
Blood loss (ml)	1250 (750, 2600)	1500 (850, 2475)	0.67	1500 (700, 3000)	1225 (800, 2000)	0.31	1750 (1150, 3000)	1200 (575, 2100)	**0.038**
ICU stay (days)	4 (3, 7)	5 (3, 7)	0.72	5 (4, 7)	4.5 (3.0, 6.0)	0.14	5.5 (4, 7.5)	4 (3, 6)	**0.041**
Hospital stay (days)	17 (13, 27)	17 (13, 25)	0.99	18 (13, 25)	17 (13, 25)	0.45	22 (15, 38)	15 (12, 22)	**0.007**
Intraoperative red blood cell transfusions (units)	2 (0, 4)	2 (0, 4)	0.37	2 (0, 5)	0 (0, 4)	**0.043**	2 (0, 4.75)	0 (0, 4)	0.056
Postoperative red blood cell transfusions (units)	2 (1, 6)	2 (0, 14)	0.69	5 (2, 16)	2 (0, 6)	**0.002**	5 (2, 16)	2 (0, 9)	**0.012**
Total red blood cell transfusions (units)	7 (2, 13)	7 (2, 18)	0.99	10 (4, 21)	4 (0, 10.5)	**0.002**	12 (4, 12)	5 (0, 12.5)	**0.006**
Number of revision surgeries	0 (0, 1)	0 (0, 1)	0.67	0 (0, 1)	0 (0, 1)	**0.038**	0 (0, 1)	0 (0, 1)	0.34

*Note:* SMI (sarcopenia) and PMRA (myosteatosis) values were obtained from 103 patients; ^31^P MRS values were obtained from 99 patients; data on posttransplant resource utilization (first column) were obtained from all transplanted patients (*N* = 105).

We did not observe a statistically significant increase in 90‐day mortality in sarcopenic patients (OR = 2.26, 95% CI: 0.21–49.45, *p* = 0.51) or in patients with abnormal MR spectra (OR = 4.40, 95% CI: 0.41–96.86, *p* = 0.23). Because there was no death within 90 days in patients without myosteatosis, relative risk cannot be calculated in this case.

### 3.4 Changes in ^31^P MR Spectra After Liver Transplantation

**FIGURE 4:**
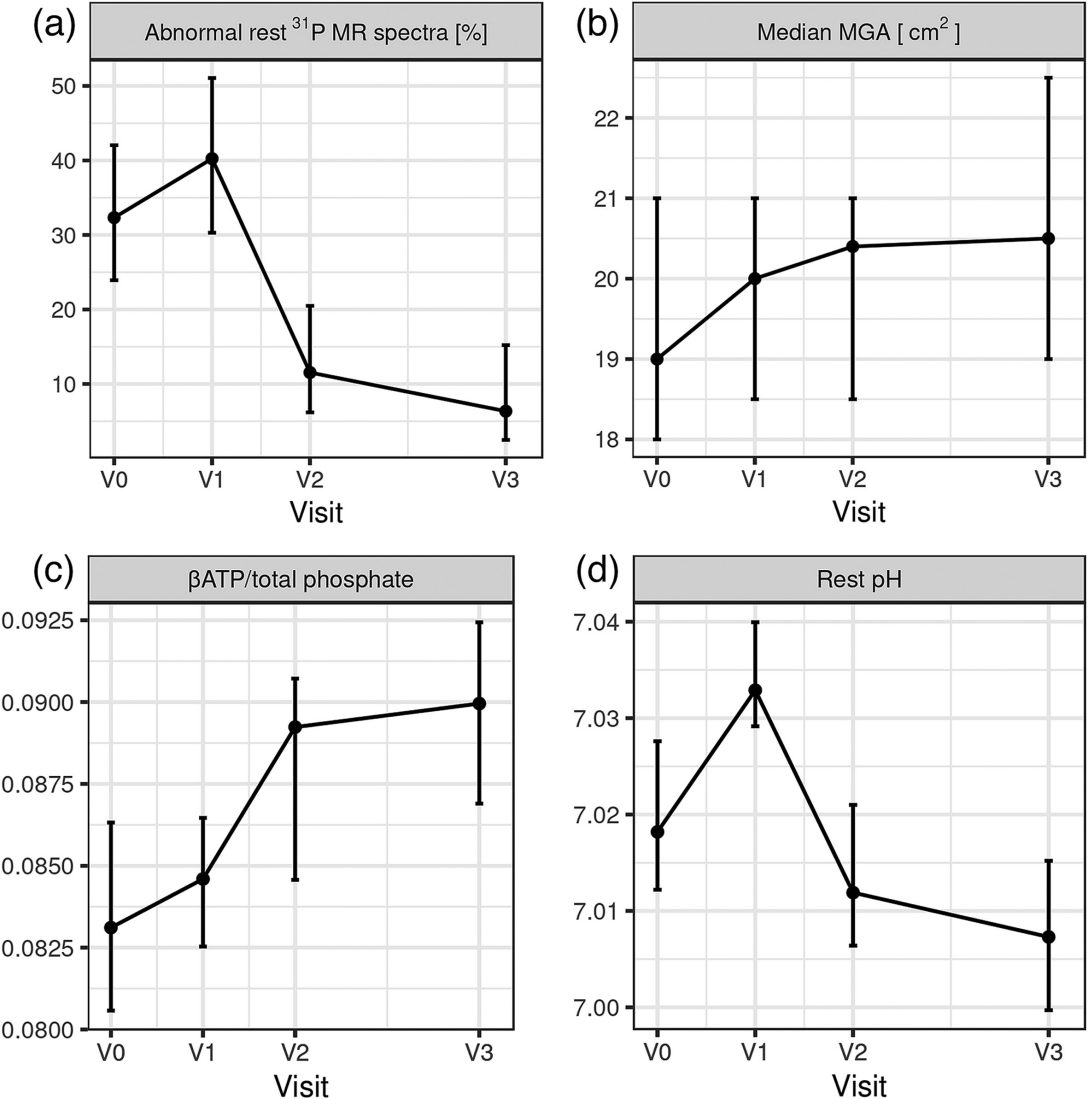
Evolution of results of ^31^P MRS of calf muscles after liver transplantation. Plots show (a) the proportion of patients with abnormal ^31^P MR spectra along with the 95% CI, (b) the area of the musculus gastrocnemius (MGA) and (c, d) the values of two variables used to define abnormal ^31^P MR spectra (pH > 7.045 or ßATP/P_tot_ < 0.074). Only data of patients who underwent LT are shown. V0: before LT (during evaluation of LT), V1: 6 months after LT, V2: 1 year after LT, V3: 2 years after LT.

In patients who underwent LT, we tracked the metabolic status of calf muscles assessed by ^31^P MRS at 6, 12 and 24 months after LT. When evaluating candidates who later underwent LT, ^31^P MRS changes in the calf muscles were identified in 32 out of 99 cases (32.3%). There was a significant improvement in the metabolic status of calf muscles 12 months after LT; only 9 out of 78 (11.5%) patients exhibited abnormal ^31^P MR spectra. This improvement was significant compared with the metabolic statuses recorded before and 6 months after LT (both *p* < 0.001). An additional decrease in the proportion of patients with abnormal ^31^P MR spectra was observed 24 months after LT (4 out of 63 patients, 6.3%). The evolution of the ^31^P MRS results after LT is shown in Figure 4a. Notably, the area of the gastrocnemius muscle had a tendency to enlarge after LT (Figure 4b).

## 4 Discussion

Our prospective study indicates that the ^31^P MR spectra of calf muscles at rest may be a novel potential prognostic marker for long‐term survival, perioperative outcomes and resource utilization in patients undergoing LT. We observed that abnormalities in ^31^P MR spectra had a higher predictive value than CT‐diagnosed myosteatosis or sarcopenia. Furthermore, our multivariable analysis suggests that ^31^P MRS may be the best predictor of patient outcomes after LT, even in the context of other readily available clinical and biochemical variables such as age, BMI or MELD score (Table 2). To our knowledge, our study is the first to describe the evolution of changes in muscle metabolism after LT. We observed a significant improvement in muscle metabolism detected by ^31^P MRS during the first year after LT.

Over the last decade, the assessment of muscle health has been incorporated into the clinical practice guidelines of medical societies devoted to the study of liver disease and nutrition [5]. Groups of investigators specializing in diverse fields of medicine and surgery, including LT, have described the measurement of sarcopenia using CT scanning or other specialist devices and explored the recent trend for employing myosteatosis to estimate muscle quality [3, 22]. The assessment of muscle wasting has long been employed as a bedside clinical approach for gauging the severity of chronic liver disease and the prognosis of candidates undergoing surgical procedures. Recently, this clinical approach has been complemented by the correlation of frailty with surgery outcomes [2]. The universal use of CT scanning prior to LT enables clinicians to measure muscle mass with a high degree of accuracy. Several methods for calculating sarcopenia based on CT images have been proposed [3, 23]. Myosteatosis involves the presence of ectopic fat, which modifies the attenuation of skeletal muscle during CT scanning [22]. The predictive capabilities of sarcopenia and myosteatosis CT scans have been investigated extensively in retrospective analyses of patients who have undergone LT, but with inconclusive results [7, 9, 22, 24, 25].

Our results suggest that ^31^P MRS results are better at predicting short‐ and long‐term outcomes after LT than myosteatosis or sarcopenia assessed by standard pretransplant CT examination. We assume that the main limitation of CT‐based assessment of skeletal muscle, especially in the context of diagnosing sarcopenia and myosteatosis, is its exclusive focus on morphological changes. In fact, it appears of little use in elucidating the functional or metabolic status of skeletal muscle, which explains why we found no correlation between the abnormal ^31^P MR spectra of the calf muscles and CT results documenting the presence of sarcopenia (SMI) or myosteatosis (PMRA). As the Euler diagram in Figure 3 shows, groups of patients with abnormal ^31^P MRS, myosteatosis and sarcopenia seem to only partially overlap. Notably, however, patients exhibiting both myosteatosis and abnormal ^31^P MR spectra had the worst outcomes of all the subgroups.

Radiation exposure is another significant limitation of the CT‐based approach. For this reason, repeat CT scans are not recommended for evaluating muscle changes after therapeutic interventions or when the condition of the patient deteriorates while on the waiting list. The major advantage of ^31^P MRS is that it allows skeletal muscle energy metabolism to be examined noninvasively, in vivo and on a repeated basis.

Abnormal resting muscle metabolism can manifest either as a relative decrease of the βATP signal intensity, representing the deterioration in the energy status of muscle cells, or as an increase in the resting intramyocellular pH value, an indicator of overall muscle cell homeostasis. In our study, both of these markers were of prognostic value. PCr, Pi and ATP metabolites are closely related to muscle cell energy turnover [26]. The numerous functions of ATP, mainly produced by the mitochondria, include muscle contraction, ion transport and the transfer of chemical energy for synthesis reactions. Quantitative studies involving healthy volunteers have demonstrated the stability of the ATP signal in these individuals, making it a reliable reference signal for other measurements [26]. Note that in patients with liver failure, the ability of cells to maintain a stable cytosolic ATP level is significantly impaired. Although an increase in resting intramyocellular pH may not be as dramatic as the pH decrease typically observed during anaerobic exercise (measured by dynamic ^31^P MRS), maintaining the optimal resting pH is crucial for the proper functioning of all biochemical processes. For instance, an increase in resting pH may highlight the reduced capacity of muscle cells to maintain normal pH values, especially when compared with the more alkaline extracellular spaces of the blood plasma and interstitium. In our study, these MRS abnormalities disappeared within the first year after LT in the majority of patients, indicating an association between MRS changes and the metabolic debilitation of patients prior to transplantation.

Based on our detailed analysis of all six variables measured by ^31^P MR spectra at rest, the only correlation of note was between the ratio of the signal of βATP to the sum of all ^31^P metabolites and SMI (Table 3 and Figure S3); none of these variables correlated with PMRA. In contrast, we found a positive correlation between SMI and the area of the gastrocnemius muscle measured by MR (Table 3). This finding seems to validate our measurements and support our conclusion that morphological and metabolic assessments relay different aspects of information on the muscle compartment. Interestingly, we found no correlation between the presence of sarcopenia and myosteatosis, which leads us to assume that these are independent variables.

Though ^31^P MRS seems to be a more logistically demanding technique requiring an MR system equipped with a multinuclear unit, ^31^P coils and spectroscopy sequences, we found that nonlocalized ^31^P MR spectra were not time‐consuming to examine (taking only 4 min to measure ^31^P MR spectra in our case) or challenging to evaluate [11].

The absence of any correlation between the presence of sarcopenia and outcomes in our study is noteworthy. A systematic review and meta‐analysis published in 2016 found that patients with sarcopenia had a 1.84 higher risk of death after LT compared with patients without sarcopenia (HR, 1.84; 95% CI, 1.11–3.05) [24]. However, in our cohort, we found no significant association between the presence of sarcopenia and the outcomes of therapeutic intervention. Indeed, our data are consistent with recently published results by groups from Aachen [7, 8, 9] and Johannesburg [25] confirming an association of myosteatosis, but not sarcopenia, with perioperative outcomes and long‐term survival after LT. In our study, we even identified a trend towards improved survival in patients with sarcopenia (Figure 2a). Without ruling out pure chance, this may be due to the negative correlation between sarcopenia and BMI (*r* = −0.48; 95% CI, −0.60 to −0.34) and the fact that a high BMI was associated with worse survival (HR, 2.63; 95% CI, 0.98–7.05; *p* = 0.046). Therefore, the better survival observed in sarcopenia patients may be attributed to their lower average BMI. In fact, had sarcopenia and BMI been combined in a single model to predict survival, the trend towards sarcopenia would have been almost completely negligible. Another possible explanation is that clinical awareness of the importance of good nutritional status influences the preselection of candidates for LT, leading to the preferential referral of those sarcopenic patients who would otherwise be considered less at risk for LT. We speculate that this particular aspect of clinical decision‐making, which involves overcompensating for the initial clinical disadvantage, explains the trend towards improved outcomes of sarcopenic candidates in our group. In fact, this phenomenon has been previously described in studies investigating the approaches of transplant programs to various risk scores [27, 28]. Our data support the premise that the evaluation of muscle metabolism may improve the differentiation of patients with muscle compartment alterations, who are consequently at high risk of short‐ and long‐term posttransplant mortality, compared with CT‐based techniques that only evaluate muscle mass or density.

Based on the evolution of ^31^P MR spectra after LT, we observed a significant improvement in the metabolic status of calf muscles 12 months after LT (Figure 4a). In addition, we observed that the gastrocnemius muscle area tended to enlarge after LT (Figure 4b). These findings may indirectly prove that changes in pretransplant skeletal muscle are associated with poor liver function and that the MRS parameters selected, which reflect the impaired metabolism of skeletal muscle, were chosen correctly.

Although the data presented in our prospective study are promising, they need to be validated in larger and, ideally, multicentre cohorts, the absence of which is the main limitation of our study. We also see value in carrying out absolute quantification of phosphorus metabolites in different muscle groups, which may provide additional insights into the metabolism of muscles in these patients.

In conclusion, our study appears to show a strong association between skeletal muscle metabolism, characterized by ^31^P MR spectra and both short‐ and long‐term survival outcomes post‐LT. Moreover, improvement of muscle metabolism within 1 year of LT was observed in most patients.

## Conflicts of Interest

The authors declare no conflicts of interest.

## Supporting information


**Figure S1** Study flowchart.
**Figure S2** Illustrative CT images of patients before LT (a) without and (b) with sarcopenia. Highlighted areas of the psoas and other abdominal muscles (green) were used to calculate the skeletal muscle index (SMI) at the L3 vertebra level. Illustrative CT images of patients before LT (c) without and (d) with myosteatosis. Highlighted areas of the psoas muscles (pink) were used to calculate the average density.
**Table S1** Indications for liver transplantation in LT candidates.
**Table S2** Tumor characteristics for transplanted patients with histologically proven hepatocellular carcinoma (HCC) based on the American Joint Commission on Cancer (AJCC) 8th edition staging system for patients with HCC. Primary tumor (T) and stage data were only obtained from 26 patients because of necrotic lesions in 2 patients due to previous transarterial chemoembolization (TACE). The last available AFP (alpha‐fetoprotein) value prior to LT is presented. Data are given as N (%) or the median (first to third quartiles).
**Table 3** Five‐year outcomes in transplanted patients based on skeletal muscle changes.
**Figure S3** Scatterplots showing relationships between (a) SMI (sarcopenia) and PMRA (myosteatosis), (b) SMI and ßATP/P_tot_ and resting pH, and (c) PMRA and ßATP/P_tot_ and resting pH.
**Figure S4** Probability of survival in LT candidates based on the presence of (a) sarcopenia, (b) myosteatosis, and (c) abnormal 31P MR spectra. Kaplan–Meier curves (step functions) with 95% confident intervals (semitransparent areas). The zero point on the *x* axis is the date of admission for the pretransplant evaluation.
