# Supplementary material for: Skeletal Muscle 31P MR Spectroscopy Surpasses CT in Predicting Patient Survival After Liver Transplantation
Source: J Cachexia Sarcopenia Muscle. 2024 Nov 23;16(1):e13635. doi: 10.1002/jcsm.13635 (PMC11670159; doi:10.1002/jcsm.13635)
Supplement: Supplementary file 1 — Figure S1 Study flowchart. Figure S2 Illustrative CT images of patients before LT (a) without and (b) with sarcopenia. Highlighted areas of the psoas and other abdominal muscles (green) were used to calculate the skeletal muscle index (SMI) at the L3 vertebra level. Illustrative CT images of patients before LT (c) without and (d) with myosteatosis. Highlighted areas of the psoas muscles (pink) were used to calculate the average density. Table S1 Indications for liver transplantation in LT candidates. Table S2 Tumor characteristics for transplanted patients with histologically proven hepatocellular carcinoma (HCC) based on the American Joint Commission on Cancer (AJCC) 8th edition staging system for patients with HCC. Primary tumor (T) and stage data were only obtained from 26 patients because of necrotic lesions in 2 patients due to previous transarterial chemoembolization (TACE). The last available AFP (alpha‐fetoprotein) value prior to LT is presented. Data are given as N (%) or the median (first to third quartiles). Table 3 Five‐year outcomes in transplanted patients based on skeletal muscle changes. Figure S3 Scatterplots showing relationships between (a) SMI (sarcopenia) and PMRA (myosteatosis), (b) SMI and ßATP/Ptot and resting pH, and (c) PMRA and ßATP/Ptot and resting pH. Figure S4 Probability of survival in LT candidates based on the presence of (a) sarcopenia, (b) myosteatosis, and (c) abnormal 31P MR spectra. Kaplan–Meier curves (step functions) with 95% confident intervals (semitransparent areas). The zero point on the x axis is the date of admission for the pretransplant evaluation. [file JCSM-16-e13635-s001.pdf]

Skeletal muscle ^{31}P MR spectroscopy surpasses CT in predicting patient survival after liver transplantation

Supplementary Material

Supplementary Figure 1 Study flowchart

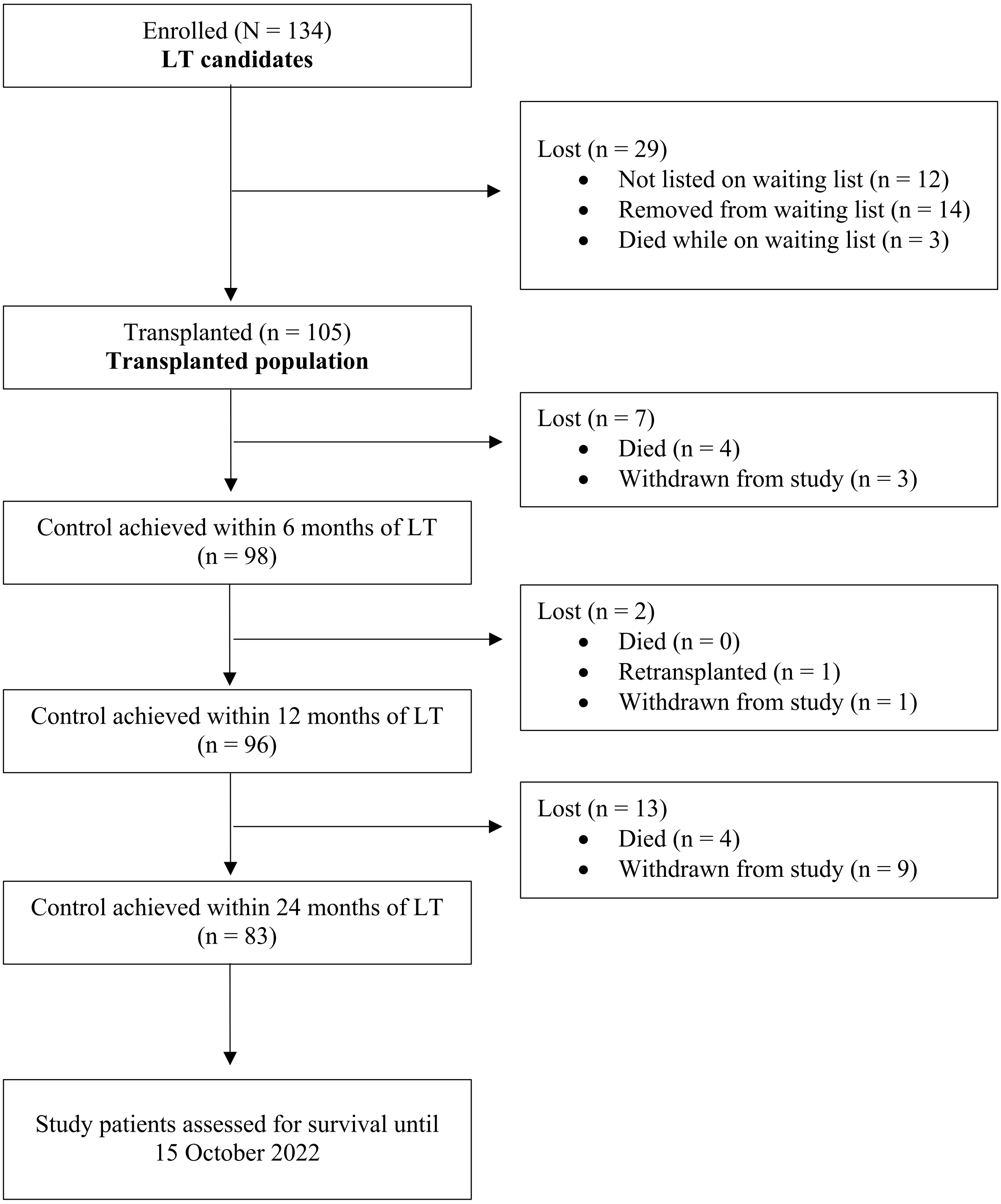

Supplementary Figure 2 Illustrative CT images of patients before LT (a) without and (b) with sarcopenia. Highlighted areas of the psoas and other abdominal muscles (green) were used to calculate the skeletal muscle index (SMI) at the L3 vertebra level. Illustrative CT images of patients before LT (c) without and (d) with myosteatosis. Highlighted areas of the psoas muscles (pink) were used to calculate the average density.

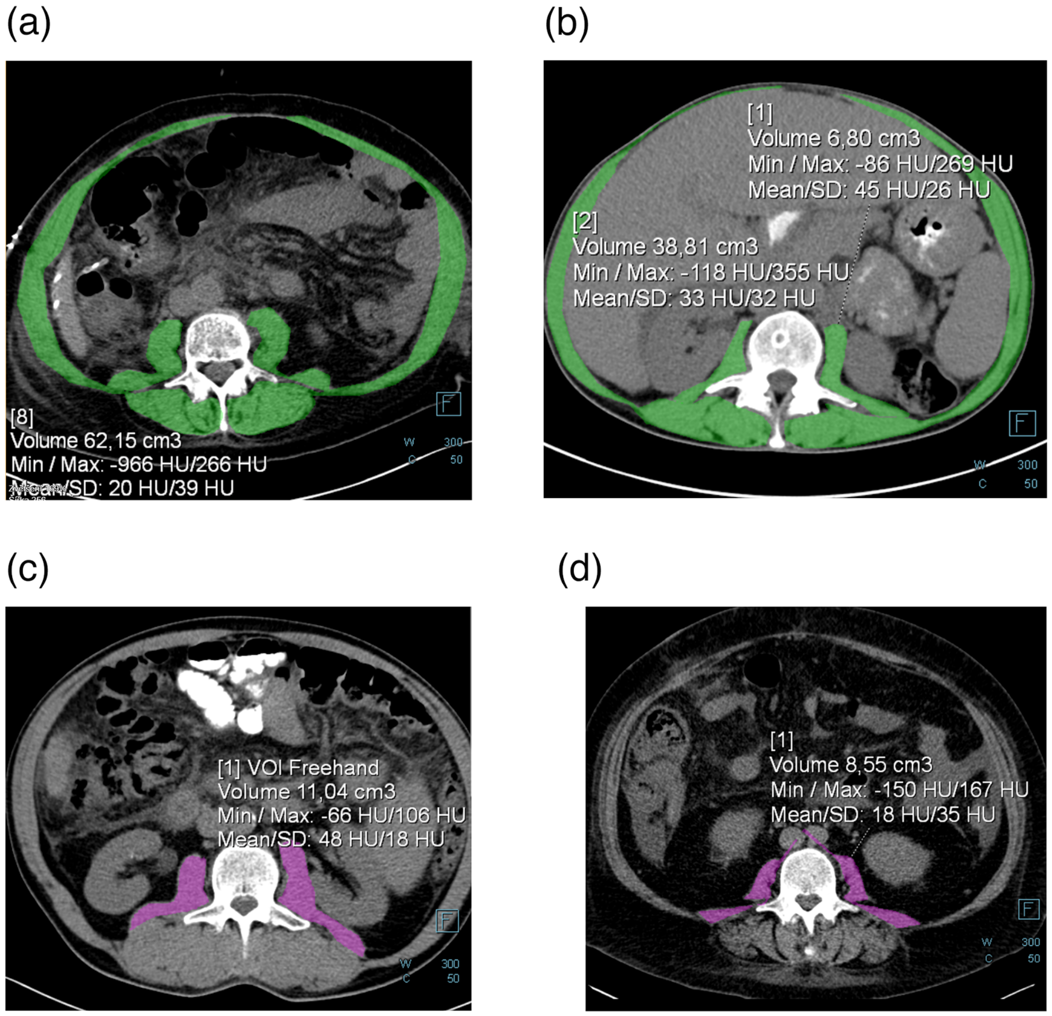

Supplementary Table 1 Indications for liver transplantation in LT candidates

**Indication for LT**	**LT candidates (N = 134)**
Alcoholic liver disease	46 (34.3%)
Cholestatic liver disease (PBC, PSC, PSC–AIH overlap syndrome)	23 (17.2%)
Hepatitis C virus	18 (13.43%)
Hepatitis B virus	9 (6.7%)
MASH	14 (10.45%)
Cryptogenic cirrhosis	6 (4.5%)
Autoimmune cirrhosis	4 (3%)
Polycystic liver disease	4 (3%)
Wilson's disease	2 (1.5%)
Neuroendocrine tumor	2 (1.5%)
Other (HCC in the noncirrhotic liver, alpha-1 antitrypsin deficiency, epithelioid hemangioendothelioma, alveolar echinococcosis, angiosarcoma, hemochromatosis)	6 (4.5%)

Of the 105 patients who underwent LT, hepatocellular carcinoma was histologically proven in 28 patients (26.7%) and cholangiocarcinoma was histologically proven in 3 patients (2.9%). Abbreviations: PBC – primary biliary cholangitis, PSC – primary sclerosing cholangitis, AIH – autoimmune hepatitis, MASH – metabolic dysfunction-associated steatohepatitis, HCC – hepatocellular carcinoma.

Supplementary Table 2 Tumor characteristics for transplanted patients with histologically proven hepatocellular carcinoma (HCC) based on the American Joint Commission on Cancer (AJCC) 8th edition staging system for patients with HCC. Primary tumor (T) and stage data were only obtained from 26 patients because of necrotic lesions in 2 patients due to previous transarterial chemoembolization (TACE). The last available AFP (alpha-fetoprotein) value prior to LT is presented. Data are given as N (%) or the median (first to third quartiles).

	**HCC presence (N = 28)**
Number of lesions	1 (1, 2)
Size of largest lesion (cm)	2.25 (1.68, 4.13)
Meeting Milan criteria	21/28 (75%)
AFP (µg/L)	6.75 (3.28, 32.98)
Histologic grade (G)	
1	6/27 (22.2%)
2	15/27 (55.6%)
3	6/27 (22.2%)
4	0/27 (0%)
Primary tumor (T)	
pT1	11/26 (42.3%)
pT2	14/26 (53.8%)
pT3	1/26 (3.8%)
pT4	0/26 (0%)
Regional lymph nodes (N)	
pNx	14/28 (50%)

pN0	14/28 (50%)
pN1	0/28 (0%)
Distant metastasis (M)	
M0	28/28 (100%)
M1	0/28 (0%)
Stage	
I	11/26 (42.3%)
II	14/26 (53.8%)
III	1/26 (3.8%)
IV	0/26 (0%)

Supplementary Table 3 Five-year outcomes in transplanted patients based on skeletal muscle changes

	**5-year survival probability (%)**	**95% CI (%)**
**With sarcopenia**	87.5	(78.6–97.4)
**Without sarcopenia**	75.9	(65.3–88.2)
**With myosteatosis**	73.3	(62.2–86.3)
**Without myosteatosis**	90.0	(82.0–98.7)
**Abnormal ^{31}P MRS**	62.5	(47.8–81.7)
**Normal ^{31}P MRS**	89.5	(82.4–97.2)
	**5-year survival probability difference [%]**	**95% CI [%]** *p*
**Sarcopenia**	11.6	(3.2 – 26.4) 0.12
**Myosteatosis**	–16.7	(–31.3 to –2.0) 0.025
**Abnormal ^{31}P MRS**	–27.0	(–45.3 to –8.6) 0.004

Supplementary Figure 3 Scatterplots showing relationships between (a) SMI (sarcopenia) and PMRA (myosteatosis), (b) SMI and ßATP/P_{tot} and resting pH, and (c) PMRA and ßATP/P_{tot} and resting pH

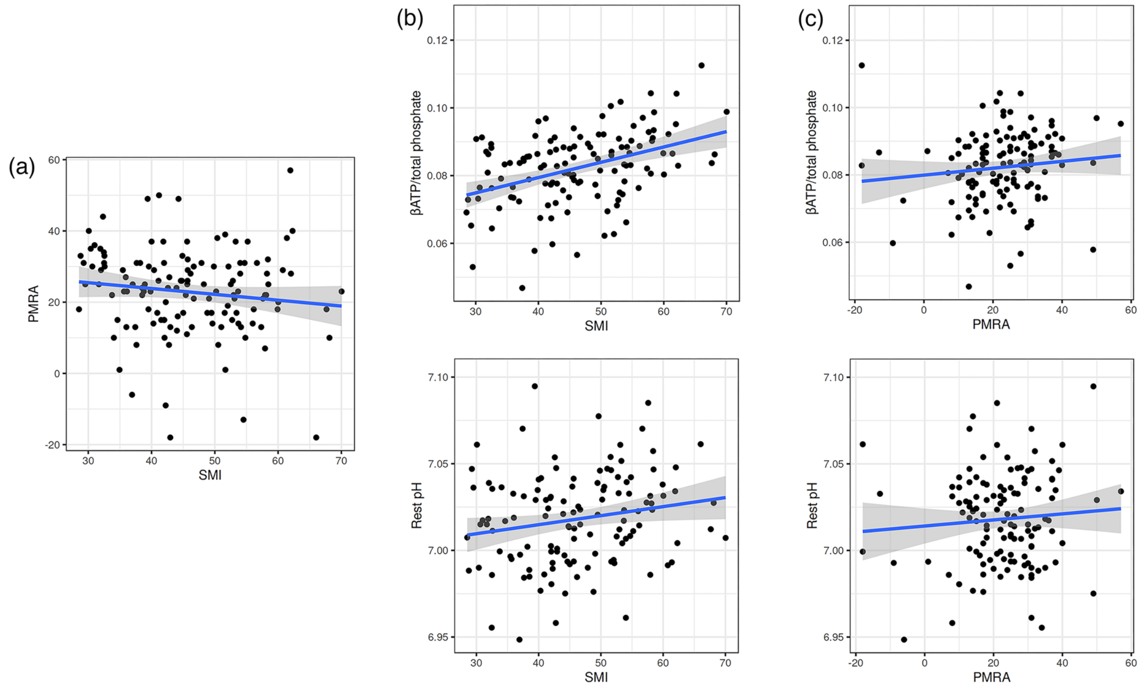

Supplementary Figure 4 Probability of survival in LT candidates based on the presence of (a) sarcopenia, (b) myosteatosis, and (c) abnormal ^{31}P MR spectra. Kaplan–Meier curves (step functions) with 95% confident intervals (semitransparent areas). The zero point on the *x* axis is the date of admission for the pretransplant evaluation.

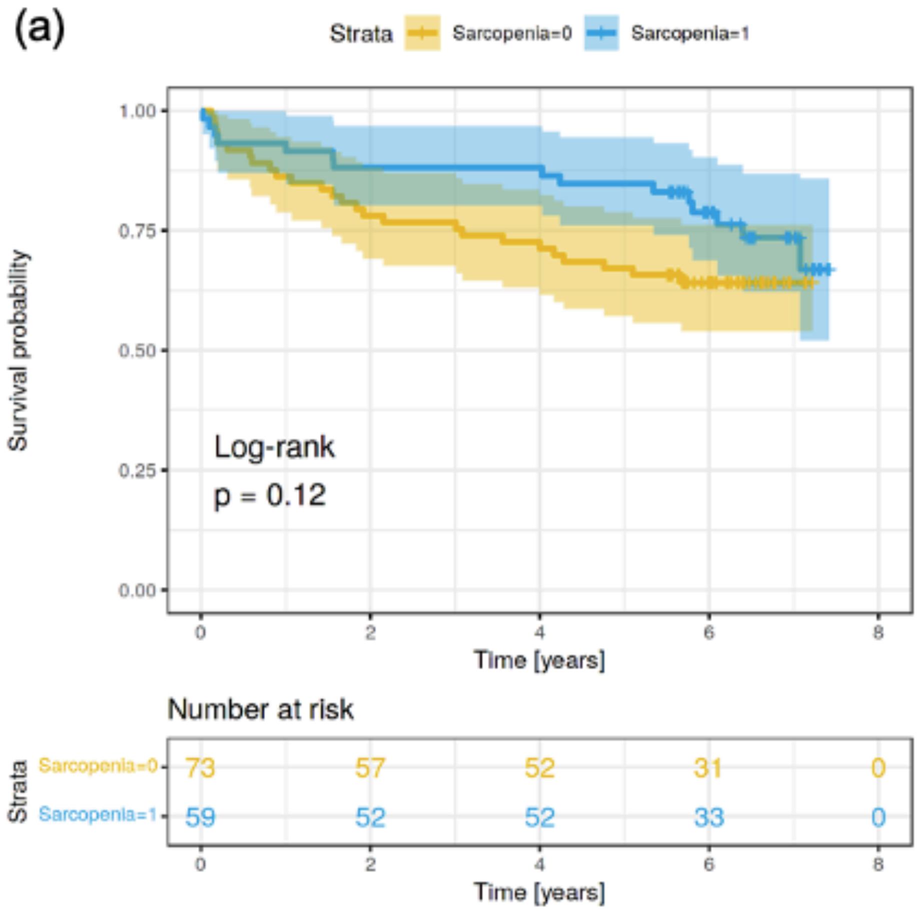

(b)

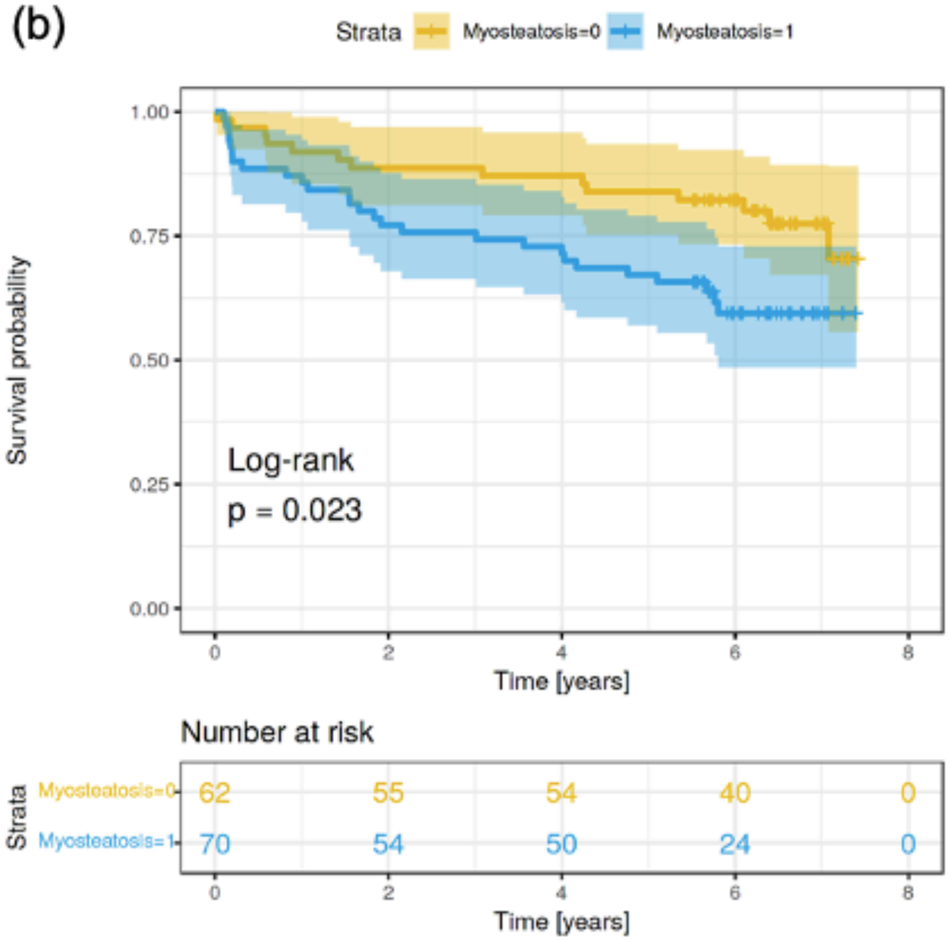
Strata
Myosteatosis=0
Myosteatosis=1
Survival probability
1.00
0.75
0.50
0.25
0.00
Log-rank
p = 0.023
0
2
4
6
8
Time [years]
Number at risk
Strata
Myosteatosis=0
Myosteatosis=1
62
55
54
40
0
70
54
50
24
0
Time [years]

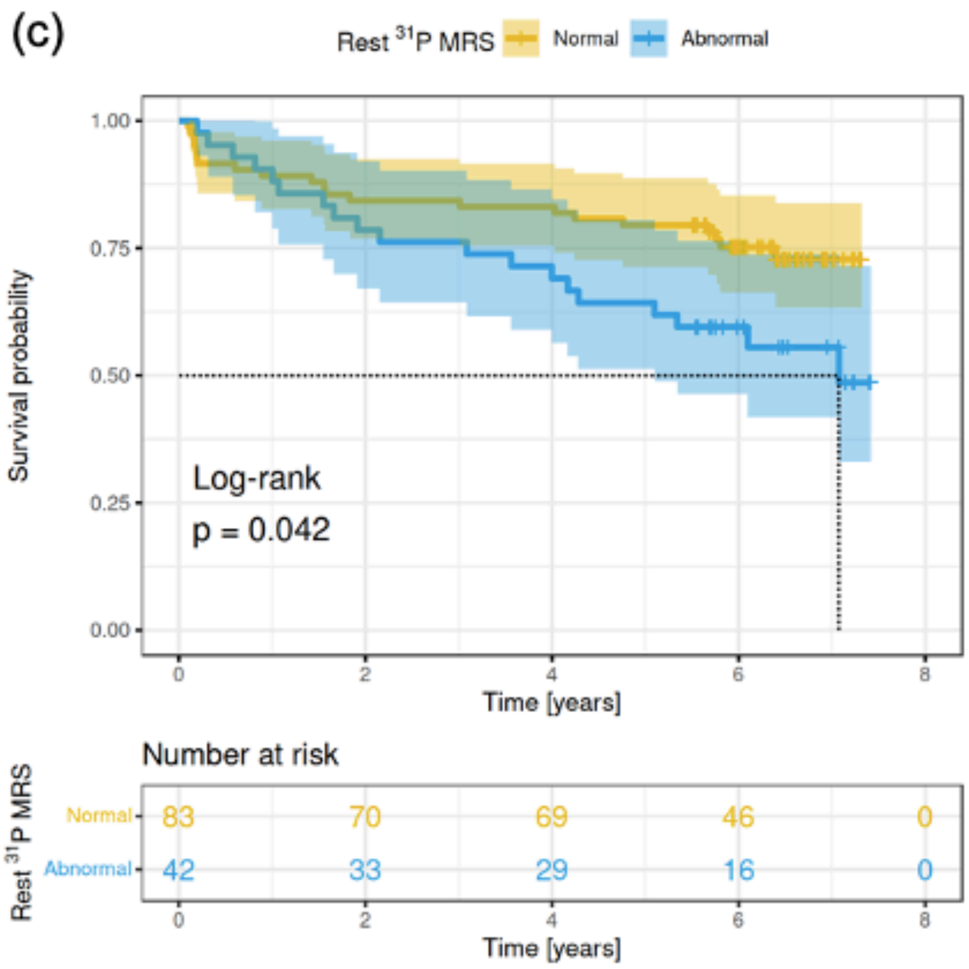
(c)
Rest ^{31}P MRS
Normal
Abnormal
Survival probability
1.00
0.75
0.50
0.25
0.00
Log-rank
p = 0.042
0
2
4
6
8
Time [years]
Number at risk
Rest ^{31}P MRS
Normal
83
70
69
46
0
Abnormal
42
33
29
16
0
Time [years]
